# A New Extension of the Binomial Error Model for Responses to Items of Varying Difficulty in Educational Testing and Attitude Surveys

**DOI:** 10.1371/journal.pone.0141981

**Published:** 2015-11-06

**Authors:** James A. Wiley, John Levi Martin, Stephen J. Herschkorn, Jason Bond

**Affiliations:** 1 Department of Family and Community Medicine and Institute for Health Policy Studies, School of Medicine, University of California San Francisco, San Francisco, California, United States of America; 2 Sociology Department, University of Chicago, Chicago, Illinois, United States of America; 3 Department of Mathematics, College of Staten Island, New York City, New York, United States of America; 4 Alcohol Research Group, Emeryville, California, United States of America; University of East Piedmont, ITALY

## Abstract

We put forward a new item response model which is an extension of the binomial error model first introduced by Keats and Lord. Like the binomial error model, the basic latent variable can be interpreted as a probability of responding in a certain way to an arbitrarily specified item. For a set of dichotomous items, this model gives predictions that are similar to other single parameter IRT models (such as the Rasch model) but has certain advantages in more complex cases. The first is that in specifying a flexible two-parameter Beta distribution for the latent variable, it is easy to formulate models for randomized experiments in which there is no reason to believe that either the latent variable or its distribution vary over randomly composed experimental groups. Second, the elementary response function is such that extensions to more complex cases (e.g., polychotomous responses, unfolding scales) are straightforward. Third, the probability metric of the latent trait allows tractable extensions to cover a wide variety of stochastic response processes.

## Introduction

In this paper we introduce a class of item response models for the analysis of response distributions derived from survey data. The simplest item response function in this class is a generalization of the binomial error model for ability testing of Keats and Lord [1]. Similar item response functions were considered briefly by Lazarsfeld [2] and Coleman [3] but not implemented as models for response data. The distinguishing characteristic of the approach taken here is that the probability of an item response is written as a function of a latent variable that can also be interpreted as a probability. The choice of a probability metric suggests the Beta density as a natural choice to model the distribution of the latent variable. Furthermore, a response function formulated in this way is easy to modify to accommodate variations in the nature and complexity of response tasks.

We first introduce the extended binomial error (EBE) model as a generalization of the binomial error model. We discuss issues of identifiability, estimation and fit, and give two examples, one for a single sample, and another for data from two independent samples, noting the similarities in fit between the extended binomial error and loglinear Rasch models. Finally, we discuss extensions for polychotomous data and for modeling responses produced by non-cumulative response functions.

The resulting class of models have special utility for the investigation of substantively important questions about the nature of response (as opposed to scoring long tests), Further, in contrast to the loglinear Rasch models that have been most influential in sociology due to the work of Duncan [4] in particular, this approach allows us to make strong assertions about the social *distribution* of the latent trait.

## The Extended Binomial Error Model

### Introducing item difficulty into the binomial error model for dichotomous responses

The binomial error model for test scores was introduced by Keats and Lord [1] [5] as a strong true score theory for dichotomous test items of more or less equal difficulty. This model is based on the assumption that the conditional distribution of the observed score given the true score (conceived as a theoretical “proportion correct” measure) is binomial, with the true score playing the role of the constant probability of a correct answer in *M* independent trials where *M* is the number of items. With no additional specifications, Keats and Lord were able to show that the first *M* moments of the true score distribution can be determined from the moments of the observed score distribution. Furthermore, they showed [6] that a linear regression of true on observed score implies and is implied by a negative hypergeometric distribution of the observed scores. They also proved that a two-parameter Beta distribution for the true scores implies a linear regression of true score on observed score and a negative hypergeometric distribution for the observed scores.

Of course, it is infrequent that we analyze a set of dichotomous items with the same levels of difficulty (see also [7]); hence Keats and Lord indirectly incorporated variations in item difficulties in their compound binomial model, which accordingly lost many of the most attractive features of the binomial error model. Some other modifications of the binomial error assumptions have been proposed [8], but these are not often implemented as IRT models.

We propose to modify the binomial error model by direct incorporation of item-difficulty parameters and by assuming a bounded proportion-like latent trait. The model assumptions allow a compact expression for the probability of any observed response pattern in terms of item-difficulty parameters and the parameters of the distribution of the latent trait, assumed to be two-parameter Beta. This expression leads in turn to an algorithm for simultaneous ML estimation of both sets of parameters. In contrast to a Rasch model, therefore, our estimation of item parameters is not separable from the estimation of person parameters; the advantage of specifying a flexible family of distributions like the Beta comes in the capacity to easily analyze factorial experiments in which either the trait, or item hardnesses, or both may be affected by treatments, as we show below.

### A Generalization

Let **x** = [*x*_1_, *x*_2_,..,*x*_M_] be a response vector for a set of *M* dichotomous items, coded so that *x*_i_ = 1 implies completion of a task related to some underlying ability or acceptance of a statement consistent with some hypothesized underlying attitude dimension and *x*_i_ = 0 otherwise. (The 1/0 coding of responses is arbitrary but useful in writing down the probabilities associated with complete response patterns.) Consider a latent variable *y*, where 0<*y*<1, and let the difficulty of the i^th^ item be *k*_i_ (*k*_i_>0 for all i). We propose the simple response function

$$\text{Pr}\left[x_{\text{i}}=1|y\right]=y^{k_{\text{i}}}$$ (1)

The conditional probability of any set of *M* responses under conditional independence is

$$\text{Pr}[x|y]=\prod_{i=1}^{n}{\left(y^{k_{i}}\right)}^{x_{i}}{\left(1-y^{k_{i}}\right)}^{\left(1-x_{i}\right)}$$ (2)

and the unconditional probability is

Pr[x]=∫Pr[x|y]φ(y)dy (3)

where ϕ(*y*) is the density of *y*.

There are a number of advantages to constructing the model using the form of Eq 1, as the latent trait may be interpreted as the probability of answering standardized (*k*_i_ = 1) item in a positive direction. However, a complication arises for the interpretation of standard errors of the *k* parameters given that they must be strictly non-negative. As is the case for other models with necessarily non-negative parameters (such as models for variances), the standard errors cannot be interpreted symmetrically; indeed, in cases of very poor fit, standard errors may imply that the true population value might quite plausibly be negative. When the model fits well, these issues are minor, but it makes sense to carry out statistical tests on the equivalence of *k* parameters not by comparison of standard errors, but by the comparison of chi-squares of nested models constraining or not constraining parameters to be equal. If the use of confidence intervals is required, one can rewrite the item response function as a double-exponential whose argument is the difference between an unbounded latent variable *ξ* and an unbounded item difficulty parameter χ_i_, where *ξ* = -log[-log(*y*)] and χ_i_ = log(*k*_i_), *ξ*, -∞< *ξ*, χ_i_ <+∞. Thus we can write

$$Pr[x_{i}=\;1|\xi ]\;=\;exp[-(exp(-(\xi -\chi_{i}))].$$ (4)

We note, however, that in contrast to a Rasch model, Eq 4 does not imply equiprobability when ξ = χ_i_; instead, Pr[*x*_i_ = 1|ξ] = .5 when (ξ-χ_i_) = -.3665. Given that Eq 1 has the more intuitive relation to a probability statement, we prefer this for the development of a family of models for stochastic response.

The choice of a probability metric suggests the Beta density as a natural choice to model the distribution of the latent variable, as it, like a probability, is defined for the interval [0,1]. Thus we propose:

$$\varphi (y)=\frac{\Gamma (a+b)}{\Gamma (a)\Gamma (b)}y^{\left(a-1\right)}{\left(1-y\right)}^{\left(b-1\right)}$$ (5)

where *a*, *b*>0, 0<*y*<1, and Γ is the complete gamma function. (We note that such a specification was suggested but not implemented by Lazarsfeld [2].) Note that E[*y*] = *a*/(*a+b*); V[*y*] = *ab*/[(*a+b*)^2^(*a+b*+1)]. For *k*_i_ = *k* for all i, this leads to the binomial error model with a Beta density for the latent trait, sometimes called the beta-binomial model [7].

It can be shown that given this density, for any *k*>0,

$$E[y^{k}]=\frac{\Gamma (a+b)\Gamma (a+k)}{\Gamma (a)\Gamma (a+b+k)}$$ (6)

Thus, for example, the unconditional probability of the unit response vector **x** = [1,1,1,…,1] is simply

$$\text{Pr}[1,1,\ldots ,1]=E\left[\text{Pr}[1,1,\ldots ,1|y]\right]=E\left[y^{\sum_{i=1}^{M}k_{i}}\right]=\frac{\Gamma (a+b)\Gamma (a+\sum_{i=1}^{M}k_{i})}{\Gamma (a)\Gamma (a+b+\sum_{i=1}^{M}k_{i})}$$ (7)

More generally, any Pr[**x**] may be written as a function of gamma functions whose arguments are the sums of the distribution parameters *a* and *b* and/or item difficulties *k*_i_.

### Representation of the Unconditional Response Probabilities

Let Pr[**x**] represent the unconditional probability of a response. For any **x**, let *I*(**x**) = {i: *x*_i_ = 0} and *C*(**x**) = {i: *x*_i_ = 1}. Let *A* be any subset of *I*(**x**), including the null set Ø and let |*A*| be the number of elements in *A*. Given these definitions, we show in Appendix A that

$$\text{Pr}[x]=\text{Pr}[x_{1},x_{2},\ldots ,x_{M}]=\sum_{A\subset I(x)}{\left(-1\right)}^{\left|A\right|}E\left[\prod_{i\in A\cup C(x)}\text{Pr}[x_{i}=1|y]\right]$$ (8)

with

$$E\left[\prod_{i\in A\cup C(x)}\text{Pr}[x_{i}=1|y]\right]=\frac{\Gamma (a+b)\Gamma (a+\sum_{i\in A\cup C(x)}k_{i})}{\Gamma (a)\Gamma (a+b+\sum_{i\in A\cup C(x)}k_{i})}$$ (9)

for the EBE model. These equations are useful for constructing joint item response functions in programming routines for ML estimation.

Initial estimates for the model parameters may be obtained from bivariate cross-classifications. Given the resulting table from any such cross-classification of the i^th^ and j^th^ items, and fixing one of the two relevant item parameters (say *k*_i_) = 1, the other three parameters *k*_j_, *a* and *b* can be estimated from the three degrees of freedom in the 2×2 table. Repeating this *M*-1 times for all cross classifications of the i^th^ item with all other items produces a complete vector of starting values. (We give the details in Appendix B.) We then use the Polak and Ribière [9] version of the Fletcher and Reeves conjugate gradient method [10] to find maximum likelihood estimates; we also use the Nelder and Mead [11] simplex methods to check that there are no preferable solutions in the area of the start values. In no case did we find that our maximum was local only.

### Scores and Score Variances from the Posterior Distribution of *y* given x

The posterior density of *y* given **x** is defined as

$$h\left[y|x\right]=\frac{\text{Pr}\left[x|y\right]\varphi \left(y\right)}{\int_{0}^{1}\text{Pr}\left[x|y\right]\varphi \left(y\right)\text{d}y}=\frac{\text{Pr}\left[x|y\right]\varphi \left(y\right)}{\text{Pr}\left[x\right]}.$$ (10)

Accordingly, the expectation of the trait score associated with a manifest response vector is just

$$E[y|x]=\int_{0}^{1}h[y|x]y\text{d}y=\frac{\int_{0}^{1}\text{Pr}[x|y]y\varphi (y)\text{d}y}{\text{Pr}[x]}.$$ (11)

We shall regard E[*y*|**x**] as an estimate of the person score corresponding to the response vector **x**. The variance of the posterior distribution serves as a measure of the precision of E[*y*|**x**] as a representation of the person score and can be calculated from the relation V[*y*|**x**] = E[*y* ^*2*^|**x**]-[E[*y*|**x**]]^2^. As discussed in Appendix A, there is a form for the score of any **x** that is similar to Eqs 8 and 9.

### Local Identifiability

Eq 8 is locally identifiable if the Jacobian matrix of the transformation from model parameters to response probabilities has full column rank for a given vector of parameter values [12]. Our numerical explorations suggest that this model, without the imposition of any additional constraints, is locally identifiable for plausible regions of the parameter space. Nevertheless, the imposition of normalizations of the form *k*_i_ = 1 for some i, providing an item-specific metric of the latent variable, produces more stable full-information maximum likelihood estimates than the unconstrained model. This is the result of the following interesting circumstance: the power of a Beta-distributed random variable is a random variable that is nearly—but not quite—distributed as Beta variable. The choice of i to fix is arbitrary in the following senses: a) the fit of the model to data is virtually independent of this choice and b) ML estimates of item parameters for all normalizations are related in a simple way. This last point deserves a brief elaboration.

Suppose for some set of data the model is normalized with respect to the h^th^ item and that ML estimates are written as *k*_i_^h^, and *a* ^h^, *b* ^h^ with *k*_i_^h^ = 1 if i = h. Let *k*_i_^h^ and *a* ^h^, *b* ^h^ be the ML estimates for normalization with respect to item h so that *k*_i_^h^ = 1 if i = h. To a close approximation, *k*_i_^j^ = *k*_i_^h^ / *k*_j_^h^. We also note that the special case in which the *b* parameter is set *a priori* to 1 is underidentified and requires an arbitrary constraint on one item parameter or, equivalently, on the *a* distribution parameter. With this constraint, there are simple closed form solutions for the ratios of item parameters to the single distributional parameter. For this case, Eq 6 simplifies to

$$\text{Pr}[x_{i}=1]=E[y^{k_{i}}]=\frac{a}{a+k_{i}}$$ (12)

and hence

$$\frac{k_{i}}{a}=\frac{\left[1-\text{Pr}[x_{i}=1]\right]}{\text{Pr}[x_{i}=1]}.$$ (13)

Thus the ratio of any item hardness to the single distribution parameter can be recreated simply as a ratio of failures to successes at the marginals.

## Illustrative Analyses of Dichotomous Responses

### Issues of Fit

We go on to apply this model to sets of dichotomous items, making comparisons to results obtained with the Rasch model [13] for the simplest cases. The overall fit of the model can be assessed using the likelihood ratio chi-square, which can also be used to test nested models we shall introduce below. But for comparison of non-nested models, we use the model selection criterion of Raftery’s BIC [14], which is equal to *L* ^2^—df*ln(*N*), where *L* ^2^ is the likelihood ratio chi-square, df represents the degrees of freedom, and *N* the number of persons in the sample. (The saturated model has a BIC of 0, any model with a negative BIC is preferred to the saturated model, and the model with the lowest BIC is preferred.) As recently emphasized by Weakliem [15], BIC is not without its drawbacks, and more rigorous implementations of Bayesian logic are now tractable [16]. However, the chief practical drawback of BIC seems to be its overly conservative nature, as it prefers more parsimonious models, and given the temptation to over-fit data sets, we think that BIC serves well as a general criterion for model comparison in which many models are fit to the same data set.

### The Single Group Case

We begin by re-analyzing the classic Army data presented by Stouffer [17] and analyzed using a Rasch model by Duncan [4] and Kelderman [18]. The data are to be found in Table 1. We present the model that results when we constrain *k*_4_ = 1; the likelihood chi-square is 17.42, with 10 degrees of freedom (p = .066). Raftery’s BIC for this model is –51.66. By contrast, the Rasch model implemented by Duncan had a likelihood ratio chi-square of 10.93 at 8 degrees of freedom, (p = .206) leading to a BIC of –44.33. In sum, the loglinear Rasch model has a closer fit but uses up more degrees of freedom; while deviations from the extended binomial error are marginally significant according classical criteria, the more parsimonious model is preferred according to the Bayesian criterion.

**Table 1:** Stouffer’s Army Data.

Item *A*	Item *B*	Item *C*	Item *D*	Frequency
0	0	0	0	229
0	0	0	1	199
0	0	1	0	52
0	0	1	1	96
0	1	0	0	25
0	1	0	1	60
0	1	1	0	16
0	1	1	1	69
1	0	0	0	16
1	0	0	1	45
1	0	1	0	8
1	0	1	1	55
1	1	0	0	10
1	1	0	1	42
1	1	1	0	3
1	1	1	1	75

Most importantly, the two models agree closely as to the positions of the different items. We find a correlation > .99 between the natural log of the extended binomial error model’s item parameters and those of the Rasch model. (Here we use results from a conditional maximum likelihood fit. Duncan’s item parameters are quite different, which we assume to be a mistake, since the order of parameter hardnesses differs not only from our Rasch analyses, but from the marginal distribution of the items. Kelderman did not report item parameters.) Inspection of the estimated scores (Table 2) from the extended binomial error (Eq 11) model shows that while there are clear score differences between patterns with the same Rasch raw score (the number of “positive” responses), the ranking of respondents with respect to posterior scores is roughly the same as the ranking with respect to raw scores. The differences between posterior expected value scores over response patterns are small compared to the posterior standard deviations for each pattern; this is to be expected given that the number of items is small.

**Table 2:** Results of Fit to Army Data.

Parameter	Value	Standard Error
***k*1**	**4.0879**	**.3124**
*k*_2_	3.4005	.2534
*k*_3_	2.5674	.1875
*k*_4_	1.0000*	-------
*a*	3.0223	.3350
*b*	1.7173	.1623

* = fixed; Log-likelihood chi-square = 17.422; df = 10; p = .066.

In this case and most others we have investigated the log linear version of the Rasch and extended binomial error models lead to similar conclusions, though latter tends to be more parsimonious and the former to fit somewhat better, as it has *M*-2 more free parameters. Nevertheless, the two need not agree. Numerical investigations demonstrate the existence of cases in which response distributions generated by the extended binomial error model parameters fit the Rasch model well (as judged by goodness-of-fit measures) but produced estimates of log-linear trait distribution parameters that violate the moment inequalities conditions of Cressie and Holland [19].

In sum, the EBE behaves similarly to the Rasch model; given an implementation involving greater distributional flexibility (the loglinear version) the Rasch model will naturally fit somewhat better at the cost of more parameters. There are, however, data that are fit by one model and not by the other. Most importantly, the probability-like metric of the latent trait allows for extensions that may be of great interest when we wish to examine the mechanisms of response processes (as opposed to scoring long tests). We go on to examine several such extensions, starting with models for independent groups under experimental conditions.

### The Multiple Group Case

The multiple group extension of the extended binomial error model permits investigation of issues related to item bias and, under certain conditions, study of group differences in the distribution of the latent trait. Let g = 1,…,*G* index groups which may be formed by partitioning a single random sample, sampling from diverse populations, or by random assignment of different item formats. To allow for differences in item and distribution parameters over these groups, we may rewrite Eqs 4 and 5 as follows:

$$\text{Pr}[x_{ig}=1|y,k_{ig}]=y^{k_{ig}}$$ (14)

$$\varphi_{g}(y)=\frac{\Gamma (a_{g}+b_{g})}{\Gamma (a_{g})\Gamma (b_{g})}y^{\left(a_{g}-1\right)}{\left(1-y\right)}^{\left(b_{g}-1\right)}$$ (15)

To illustrate, we take data from a national telephone survey of Italian adults 18–69 years old that was conducted in April and May, 1994 [20]. The survey was part of a study of regional and ethnic prejudice in Italy and included a series of items dealing with stereotypical beliefs about Africans and East Europeans living in Italy. Each respondent was assigned at random to a set of questions targeted at one of three immigrant groups: a) North Africans, such as Moroccans, Tunisians, or Algerians (probability = .25); b) Africans from regions of Central Africa, such as Senegal and Somalia (probability = .25); and c) Eastern Europeans, such as Poles, Albanians, or Slavs (probability = .5). For each target group, the respondent was presented with a statement incorporating a positive or negative adjective pertaining to the group, e.g.: “do you agree that most of them are complainers? (they try to make others feel sorry for them)”. The interviewer then asked respondents “Do you agree strongly, agree somewhat, disagree somewhat, or disagree strongly with this description?”

For our example, we selected responses to items incorporating four negative adjectives which roughly translate into “selfish”, “slackers”, “violent”, and “complainers”, combined the North and Central African target groups into a single target group (based on similarity of the marginal distributions), and dichotomized the responses into “Agree” (coded 1) and “Disagree” (coded 0). The response distribution for *N* = 2001 respondents in the 1994 survey is shown in Table 3.

**Table 3:** 1994 Italian Survey: Stereotype Data (Sniderman, et al., 1995).

Item 1	Item 2	Item 3	Item 4	Immigrants are African	Immigrants are East European
0	0	0	0	342	361
0	0	0	1	164	129
0	0	1	0	35	32
0	0	1	1	44	47
0	1	0	0	53	41
0	1	0	1	60	51
0	1	1	0	18	20
0	1	1	1	51	38
1	0	0	0	35	45
1	0	0	1	39	47
1	0	1	0	9	20
1	0	1	1	28	27
1	1	0	0	11	10
1	1	0	1	39	36
1	1	1	0	13	17
1	1	1	1	66	73
			**Totals**	**1007**	**994**

As an exercise, we can use these data to determine whether the responses are consistent with the hypothesis of an underlying trait of prejudice or hostility to minorities, and if so, if Africans and Eastern Europeans are perceived identically. If there are differences in the perception of Africans and Eastern Europeans, we can determine whether there is still a single trait of “prejudice” with perhaps different thresholds for attributing negative characteristics to Africans and Eastern Europeans, or whether there seem to be different latent traits involved when it comes to judging members of the two target groups.

Table 4 presents fit statistics for selected models for these data. The first model corresponds to Eqs 14 and 15, only adding the constraint *k*_3g_ = 1 for g = 1,2 (the reason the third item is chosen will become clear below). This model fits quite well, generating a likelihood ratio chi-square of 15.93 with 20 degrees of freedom (p = .721). Models 2 and 3 impose substantively important constraints. Model 2 sets *k*_ig_ = *k*_i_ for all i; it is a test of identical item meanings (semantic invariance) that allows the choice of target group to evoke different traits (e.g., degree of hostility to Africans or degree of hostility to Eastern Europeans). This sort of model might be used to examine item bias across two non-experimental groups; if this model failed to fit one could attempt to see if there were particular items that had different hardnesses across groups.

**Table 4:** Results of Fitting Models to Italian Data.

Model	Parameters Constrained To Be Identical Across Groups	Likelihood Ratio Chi-Sq	df	Probability
**1**	*k*3*	**15.93**	**20**	**.721**
**2**	*k*1, *k*2, *k*3*, *k*4	**26.24**	**23**	**.290**
**3**	*k*3*, *a*, *b*	**16.55**	**22**	**.788**
**4**	*k*2, *k*3*, *a*, *b*	**18.56**	**23**	**.726**
**5**	*k*2, *k*3*, *k*4, *a*, *b*	**21.49**	**24**	**.610**
**6**	*k*1, *k*2, *k*3*, *k*4, *a*, *b*	**27.61**	**25**	**.326**

* = fixed parameter

Given that our groups are the results of experimental treatments, however, we may instead begin by assuming identity of the distribution of the latent trait. Model 3 sets *a*_g_ = *a* and *b*_g_ = *b*; given the normalization *k*_3g_ = 1 for g = 1,2 this is equivalent to saying that there is only one trait of overall prejudice, but the items (except for the third) can have different hardnesses depending on the target group.

Given model 1, model 2 must clearly be rejected as the difference in chi-square is significant (10.31 at 3 df, p = .016). Given model 1, however, loss of fit due to the constraints associated with model 3 is insignificant (chi-square of .62 at 2 df, p = .733). (The results here are not independent of the choice of item that is fixed; setting *k*_3g_ = 1 for g = 1, 2 resulted in the lowest chi-square for model 3 and was hence also used for models 1 and 2.) Further, model 4 demonstrates that the location of item 2 can also be equated for the two groups (the chi-square difference of 2.01 between models 3 and 4 is insignificant at 1 df with p = .156), although models 5 and 6 demonstrate that this is not true of items 1 and 4. Inspection of the item parameters demonstrates that the item locations are more spread out on the underlying continuum when the target group is Africans than when the target is Europeans. Such a result is consistent with the interpretation that there is a single trait of out-group hostility among the respondents, but that Italians have a more differentiated stereotype of Africans than they do of Eastern Europeans.

## Other Extensions

### Generalization To Ordered Polychotomies

The generalization of the extended binomial error model to ordered polychotomies relies on a standard threshold parameterization for ordered categories that was developed in an IRT context by Edwards and Thurstone [21] and also for regression analysis of ordered categories (see, for example, [22–24]). Let j = 1,…, *J* represent the *a priori* order of the response categories for the i^th^ item where j = 1 is “low” and j = *J* is “high”. We denote the hardness of each of these response categories as *k*_i,j_, where *k*_i,1_<*k*_i,2_< ….< *k*_i,J-1._ By analogy with Eq 1 for dichotomous responses, we write the probability of that a response fall into category j *or higher* as

$$\text{Pr}\left[x_{i}\geq j|y\right]=y^{k_{i,j}}.$$ (16)

For a fixed *y* value, the probabilities thus defined diminish as j increases. Given this definition, the probabilities of the intermediate response categories are calculated as differences between the probabilities associated with adjacent dichotomizations. This then implies that the model for ordered categories can be written as follows:

$$\text{Pr}\left[x_{i}=j|y\right]=y^{k_{i,j}}-y^{k_{i,j+1}}\;$$ (17)

where we set the second term to zero for *j* = *J*.

The model given as Eq 17 has a number of welcome features. First, the probability function for any category j is single-peaked; if we denote its maximum y*_j_ then (j < k) ⇔ (y*_j_ < y*_k_); y*_1_ = 0 and y*_J_ = 1. Finally, because this is a threshold model, it satisfies the two principles Jansen and Roskam [25] called the *joining assumption* (the probability of an aggregated response category is the sum of the probabilities of its constituent, usually adjacent, response categories) and *ξ-equivalence* (the latent traits embedded in aggregated and disaggregated versions of the item response functions are identical or are related by an admissable transformation). In contrast, the most methodologically tractable generalizations to the polychotomous case in the Rasch model (the rating scale and partial credit models and their relatives [26]) do not satisfy these criteria which means that a model that fits the polychotomous data may not fit dichotomous or otherwise collapsed data [27–30]. (The graded response model [31] does satisfy the collapsing conditions but is somewhat more complex.)

### An Illustration of the Model for Ordered Polychotomies

To illustrate the application of the polychotomous model to social data, we use a small example drawn from the 2000 U.S. National Alcohol Survey, a cross-sectional national probability household survey on alcohol use and problems [32]. Table 5 shows the response distribution pertaining to a cross-classification of two 4-category items dealing with reasons for abstention from alcohol in a sample of 547 women aged 18–29. They were asked “how important to you are each of the following reasons for abstaining from alcohol beverages or being careful about how much you drink.” The two reasons represented in Table 6 are “drinking is bad for your health” and “drinking can get you sick” and the response categories for both reasons are “not a reason at all,” “not an important reason,” “somewhat important” and “very important.” In this illustration, the category response functions are written so that high values are associated with responses indicating the reasons stated are important for decisions to abstain or to be careful about drinking. Thus *y* ^k1,1^ is the conditional probability, given *y*, that a respondent will regard the “drinking is bad for your health” as “an important reason” for abstaining or being careful about drinking.

**Table 5:** National Alcohol Survey Data.

Item 1	Item 2	Frequency
1	1	9
1	2	3
1	3	7
1	4	1
2	1	4
2	2	3
2	3	7
2	4	6
3	1	15
3	2	19
3	3	65
3	4	51
4	1	15
4	2	14
4	3	80
4	4	248
	**Totals**	**547**

Response categories: 1 ‘Not a reason’, 2 ‘Not an Important Reason’, 3 ‘Somewhat Important Reason’, 4 ‘Very Important.’ Item 1: “Drinking is bad for your health”; item 2: “Drinking can get you sick.”

**Table 6:** Results of Fitting Models to Data in Table 5.

Parameter	Estimate	Standard Error
*k*_1,1_	1.000*	--
*k*_1,2_	0.132	0.021
*k*_1,3_	0.062	0.014
*k*_2,1_	1.565	0.177
*k*_2,2_	0.301	0.042
*k*_2,3_	0.141	0.025
*a*	1.112	0.194
*b*	0.598	0.097

*fixed; Log-likelihood ratio chi-squared value = 12.78; df = 8; p>0.173

Table 6 shows the parameter estimates and standard errors for fitting the extended binomial error model for ordered polychotomies to these data. As judged by the likelihood ratio chi-squared value (12.78, 8df), the fit of the model to these data is acceptable. The beta distribution generated by *a* = 1.112 and *b* = 0.598 is skewed toward high values of *y* implying that the majority of respondents consider both reasons for abstinence to be important. A comparison of the category threshold parameters indicates that “bad for your health” is considered a more compelling reason for abstinence than “getting sick” among the young adult women in the national sample.

### Single-Peaked Response Functions

This threshold formulation can also be adapted to model response processes in which subjects are more likely to give a positive response to some item if that item’s location is near their own on the latent trait. Following the method of Andrich and Luo [33–36], each item is turned into a trichotomy, with two failure regions (the item is too far above the subject for her to answer in a positive direction and the item is too far below her), with the intermediate leading to a positive response. However, there is an alternative approach that is sufficiently flexible to handle a variety of empirical cases.

Here we approximate the response function in question for some set of such dichotomous items as follows:

$$\text{Pr}[x_{i}=1|y,k_{i}]=\alpha_{i}y^{k_{i}}(1-y^{k_{i}})$$ (18)

which is a unimodal function achieving its maximum when *y* ^ki^ = .5, which is equivalent to ξ = χ_i_ in an unbounded metric if we again define χ_ɩ_ = log(*k*_i_), and *ξ* = -log[-2log(*y*)]. The leading *α*_i_ may be considered akin to a discriminating ability parameter for cumulative models, or it may be considered an overall normalization factor if constrained *α*_i_ = *α* for all i; it may also be fixed in advance, such that (for example) the predicted probability of a positive response is 1 when ξ = χ_i_. Again, there is a parsimonious representation of the unconditional probability of any vector **x** for any set of parameter values **h** = (*a*,*b*,*α*_1_, … *α*_M_, *k*_1_,…,*k*_M_) (see Appendix A).

## Conclusion

In sociology the loglinear Rasch model has become the most widely known and used IRT model in sociology [37–41]. There are two reasons for this popularity. First, Duncan [4] argued that its indifference to the distribution of the latent trait made it a better scientific instrument than the covariance-based methods that dominate social modeling. Second, the Rasch model’s parameters turned out to be estimable with a very simple loglinear approach [18, 42]. This approach treats the distribution of the latent variable as a set of nuisance parameters—the total score preserves all useful information in grading respondents, and the item hardnesses can be estimated without further investigation of the distribution of the latent trait.

But preserving a metric representation of the trait aids the investigation of the response process. While other IRT models, including the Rasch model, can be adapted in this way, the model presented here allows for a wide-range of substantively important extensions to be modeled rather simply, due to the combination of a) a latent trait that can be interpreted as a probability, b) a simple family of response functions that can model dichotomous, ordered polychotomous, and unfolding-type responses, and c) a flexible two-parameter Beta distribution for the latent trait. Such a family of models can be particularly useful in the analysis of experiments that involve changes in wording, response formats, and item order.

## Appendix A

A compact representation of the predicted response probabilities for any value of the parameters can be derived from the exclusion-inclusion theorem (see, e.g., [43], 72f). Let the vector **h** = (*a*,*b*,*k*_1_,…,*k*_M_) represent the model parameters and Pr[**x**|**h**] the unconditional probability of a response for a given **h**. For any **x**, let *I*(**x**) = {i: *x*_i_ = 0} and *C*(**x**) = {i: *x*_i_ = 1). Let *A* be any subset of *I*(**x**), including the null set **Ø** and let |*A*| be the number of elements in *A*. Now since the responses to each item are assumed to be independent conditional on the value of the latent trait, we can write Pr[**x**|**h**] in product form, where the expectation is taken with respect to the distribution of the latent trait:

$$\begin{array}{l} \text{Pr}[x|h]=\text{Pr}[x_{1},x_{2},\ldots ,x_{N}]=E\left[\text{Pr}[x_{1},x_{2},\ldots ,x_{N}|y]\right]=E\left[\prod_{i=1}^{N}\text{Pr}[x_{i}|y]\right]\\ =E\left[\prod_{i\in C(x)}\text{Pr}[x_{i}=1|y]\prod_{i\in I(x)}\left(1-\text{Pr}[x_{i}=1|y]\right)\right]\\ =E\left[\prod_{i\in C(x)}\text{Pr}[x_{i}=1|y]\sum_{A\subset I(x)}{\left(-1\right)}^{\left|A\right|}\prod_{i\in A}\text{Pr}[x_{i}=1|y]\right]\\ =E\left[\sum_{A\subset I(x)}{\left(-1\right)}^{\left|A\right|}\prod_{i\in A\cup C(x)}\text{Pr}[x_{i}=1|y]\right]=\sum_{A\subset I(x)}{\left(-1\right)}^{\left|A\right|}E\left[\prod_{i\in A\cup C(x)}\text{Pr}[x_{i}=1|y]\right] \end{array}$$ (A.1)

This representation is valid for all latent trait models for dichotomous responses that assume conditional independence. For the extended binomial error model, we make the following substitution:

$$E\left[\prod_{i\in A\cup C(x)}\text{Pr}[x_{i}=1|y]\right]=\frac{\Gamma (a+b)\Gamma (a+\sum_{i\in A\cup C(x)}k_{i})}{\Gamma (a)\Gamma (a+b+\sum_{i\in A\cup C(x)}k_{i})}$$ (A.2)

The response pattern score and score variance can be expressed similarly. Written in terms of the model parameters, the score E[*y*|**x**] is given as:

$$E[y|x]=\frac{\sum_{A\subset I(x)}{\left(-1\right)}^{\left|A\right|}\frac{\Gamma (a+b)\Gamma (a+1+\sum_{i\in A\cup C(x)}k_{i})}{\Gamma (a)\Gamma (a+b+1+\sum_{i\in A\cup C(x)}k_{i})}}{\sum_{A\subset I(x)}{\left(-1\right)}^{\left|A\right|}\frac{\Gamma (a+b)\Gamma (a+\sum_{i\in A\cup C(x)}k_{i})}{\Gamma (a)\Gamma (a+b+\sum_{i\in A\cup C(x)}k_{i})}}.$$ (A.3)

Finally, there is a parsimonious representation of the unconditional probability of any vector **x** for any set of parameter values in the unfolding-type model of Eq 19. With *I*(**x**) and *C*(**x**) defined as above, and *A* any subset of *I*(**x**), let *B* be any subset of the union of this *A* with *C*(**x**), and let |*B*| be the number of elements in *B*. Then it can be shown that

$$P[x|h]=\sum_{A\subset I(x)}{\left(-1\right)}^{\left|A\right|}\left\{\left(\prod_{i\in A\cup C(x)}\alpha_{i}\right)\sum_{B\subset \{A\cup C(x)\}}{\left(-1\right)}^{\left|B\right|}E\left[y^{\left(\sum_{i\in A\cup C(x)}k_{i}+\sum_{i\in B}k_{i}\right)}\right]\right\}$$ (A.4)

For the expectation involving *y* we make the following substitution

$$E\left[y^{\left(\sum_{i\in A\cup C(x)}k_{i}+\sum_{i\in B}k_{i}\right)}\right]=\frac{\Gamma (a+b)\Gamma (a+\sum_{i\in A\cup C(x)}k_{i}+\sum_{i\in B}k_{i})}{\Gamma (a)\Gamma (a+b+\sum_{i\in A\cup C(x)}k_{i}+\sum_{i\in B}k_{i})}$$ (A.5)

and algorithms related to those discussed above can be used to obtain simultaneous estimates of score and distribution parameters.

## Appendix B

The cross-classification of responses pertaining to any two items i and j generates a 2 by 2 table of population proportions. Consider three summary measures that are sufficient to reconstruct the marginal and interior probabilities of this table: Pr[*x*i = 1, *x*j = 1], Pr[*x*i = 1], and Pr[*x*j = 1]. (Note that here, Pr[] indicates *observed* probabilities.) Under the model represented by Eqs 1 and 5, these have following structure:

$$\text{Pr}\left[x_{i}=1,\;x_{j}=1\right]=E\left[y^{k_{i}+k_{j}}\right]=\frac{\Gamma \left(a+b\right)\Gamma \left(a+k_{i}+k_{j}\right)}{\Gamma \left(a\right)\Gamma \left(a+b+k_{i}+k_{j}\right)}$$ (B.1)

$$\text{Pr}\left[x_{i}=1\right]=E\left[y^{k_{i}}\right]=\frac{\Gamma \left(a+b\right)\Gamma \left(a+k_{i}\right)}{\Gamma \left(a\right)\Gamma \left(a+b+k_{i}\right)}$$ (B.2)

$$\text{Pr}\left[x_{j}=1\right]=E\left[y^{k_{j}}\right]=\frac{\Gamma \left(a+b\right)\Gamma \left(a+k_{j}\right)}{\Gamma \left(a\right)\Gamma \left(a+b+k_{j}\right)}$$ (B.3)

These equations cannot be solved uniquely to obtain *a*,*b*, *k*i, and *k*j. However, if we set *k*i = 1 (in effect, normalizing the model by setting *y* = Pr[*x*i = 1|*y*]) and use Γ(s+1) = sΓ(s), we get

$$\text{Pr}\left[x_{i}=1,\;x_{j}=1\right]=\frac{\Gamma \left(a+b\right)\left(a+k_{j}\right)\Gamma \left(a+k_{j}\right)}{\Gamma \left(a\right)\left(a+b+k_{j}\right)\Gamma \left(a+b+k_{j}\right)}$$ (B.4)

$$\text{Pr}\left[x_{i}=1\right]=\frac{\Gamma \left(a+b\right)a\Gamma \left(a\right)}{\Gamma \left(a\right)\left(a+b\right)\Gamma \left(a+b\right)}=\frac{a}{\left(a+b\right)}$$ (B.5)

It follows that

$$\frac{\text{Pr}\left[x_{i}=1,\;x_{j}=1\right]}{\text{Pr}\left[\;x_{j}=1\right]}=\frac{a+k_{j}}{a+b+k_{j}}$$ (B.6)

and with manipulation we get

$$\frac{k_{j}}{a+b}=S_{j}$$ (B.7)

where

$$S_{j}=\frac{\text{Pr}\left[x_{i}=1,\;x_{j}=1\right]-\text{Pr}\left[\;x_{i}=1\right]\text{Pr}\left[\;x_{j}=1\right]}{\text{Pr}\left[\;x_{j}=1\right]-\text{Pr}\left[x_{i}=1,\;x_{j}=1\right]}$$ (B.8)

for all i and j. Thus, for each item other than the i^th^, its cross-classification with *x*_i_ gives us its ratio to *a* + *b*.

To construct an initial estimate of *a* + *b*, which we will denote Θ for brevity, we take advantage of the fact that from Eq B.3,

$$\text{Pr}\left[\;x_{j}=1\right]=\frac{\Gamma (\Theta )\Gamma \left(\Theta \left(\text{Pr}\left[\;x_{i}=1\right]+S_{j}\right)\right)}{\Gamma \left(\Theta \left(\text{Pr}\left[\;x_{i}=1\right]\right)\right)\Gamma \left(\Theta \left(S_{j}+1\right)\right)}$$ (B.9)

and hence

$$\text{Pr}\left[\;x_{j}=1\right]-\frac{\Gamma \left(\Theta \right)\Gamma \left(\Theta \left(\text{Pr}\left[\;x_{i}=1\right]+S_{j}\right)\right)}{\Gamma \left(\Theta \left(\text{Pr}\left[\;x_{i}=1\right]\right)\right)\Gamma \left(\Theta \left(S_{j}+1\right)\right)}=0$$ (B.10)

For admissible values of Pr[*x*i = 1], Pr[*x*j = 1], and Sj, Eq B.10 has a single positive root Θ. When the model describes the data perfectly, the *M*-1 equations corresponding to each j (j ≠ i) should yield the same root. For actual data, we average our derived values of Θ. We then substitute this value in Eq B.7 to get initial values of *k*j, j ≠ i. Our starting estimate of the parameter *a* can be recovered from the product of (*a* + *b*) and Pr[*x*i = 1] (given that, by construction, *k*i = 1), and that of *b* from *b* = Θ—*a*.
