# Correction: A New Extension of the Binomial Error Model for Responses to Items of Varying Difficulty in Educational Testing and Attitude Surveys

**DOI:** 10.1371/journal.pone.0144563

**Published:** 2015-12-03

**Authors:** James A. Wiley, John Levi Martin, Stephen J. Herschkorn, Jason Bond

There is an error in the Funding section. The complete, correct funding information is as follows:

Dr. Bond's contribution was supported in part by NIAAA-funded Center Grant P50 AA005595.
